# Analysis of Ablation Curves to Predict Transmural Lesions Using an Epicardial Bipolar Irrigated Radiofrequency Clamp in Pigs and Humans

**DOI:** 10.1177/15569845261448874

**Published:** 2026-09-06

**Authors:** Luca Aerts, Julie Norup Hertel, Ben J.M. Hermans, Justin G.L. Luermans, Sevasti-Maria Chaldoupi, Ronald Lans, Arnela Saljic, Jos G. Maessen, Thomas Jespersen, Dominik Linz, Bart Maesen

**Affiliations:** 1 Department of Cardiothoracic Surgery, Maastricht University Medical Centre, The Netherlands; 2 Cardiovascular Research Institute Maastricht (CARIM), Maastricht University, The Netherlands; 3 Department of Biomedical Sciences, University of Copenhagen, Denmark; 4 Department of Cardiology, Maastricht University Medical Centre, The Netherlands; 5 Medtronic Inc., Minneapolis, MN, USA

**Keywords:** transmurality, atrial fibrillation, ablation, irrigated radiofrequency, RF ablation, RF

## Abstract

**Objective:**

It is currently not known at what time point a lesion made with the bipolar irrigated radiofrequency clamp (Cardioblate™ Gemini-S, Medtronic, Galway, Ireland) is transmural. This study aimed to explore typical patterns of ablation curves to potentially predict the transmurality of ablation lesions created with the Gemini-S clamp during atrial fibrillation (AF) ablation.

**Methods:**

Ablations were performed on the atria and ventricles of 4 pigs. After ablation, the animals were sacrificed, and macroscopic evaluation was performed to identify the transmurality of the lesions. Typical patterns of ablation curves predicting transmurality were retrospectively identified. Second, a retrospective analysis of typical patterns in human ablation curves was performed to identify potential additional parameters associated with transmurality. Furthermore, the typical patterns identified in the pig experiments were retrospectively analyzed in human ablation curves.

**Results:**

Three typical patterns of transmurality were identified in the ablation curves in pigs. Type I was a sharp impedance rise, type II was 3 power drops of at least a 2-energy-level decline in 1 ablation cycle, and type III was a power drop (<11 s) with subsequent hovering of power levels between 15 and 25 W. In 11 hybrid AF ablation patients, 272 applications were performed via the left (41.9%) and right (58.1%) side. Of these ablations, 46.3% were classified as transmural based on the 3 typical patterns of transmurality identified in the pig experiments.

**Conclusions:**

Three typical patterns associated with transmurality have been identified using the Gemini-S clamp. These preliminary types need to be tested and validated in the clinical setting during hybrid AF ablation.

## Graphical abstract

**Graphical Abstract:**
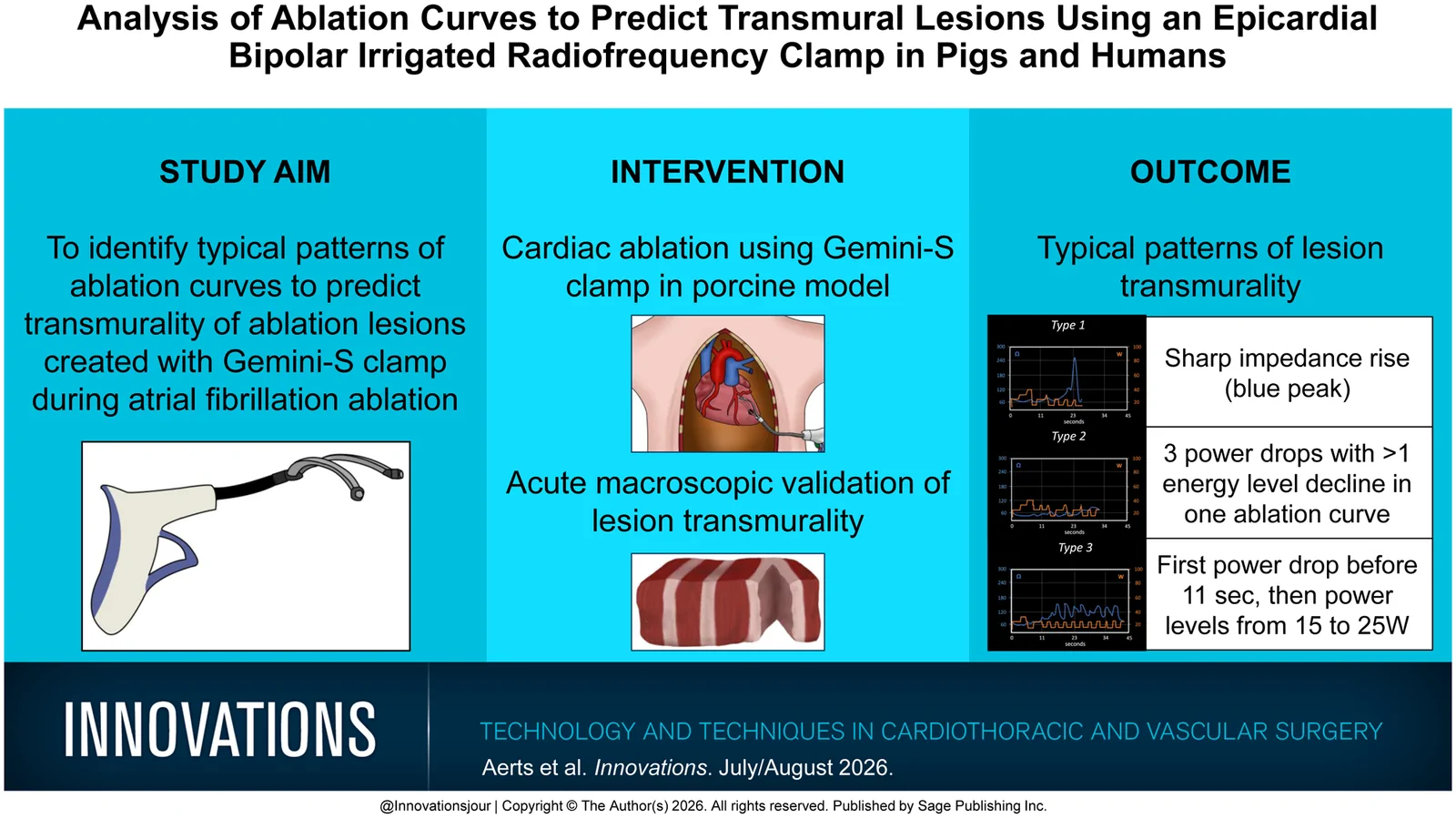
This is a visual representation of the abstract.

**Central Message**

Three recurring ablation curve patterns associated with transmurality were identified in pigs. Similar patterns were observed in retrospective human data, suggesting possible relevance for clinical interpretation. These findings are exploratory and require further validation before integration into real-time ablation guidance.

## Introduction

The goal of atrial fibrillation (AF) ablation is restoring sinus rhythm by achieving long-lasting transmural lesions.^1^ Pulmonary vein isolation (PVI) is the cornerstone of this therapy, as the pulmonary veins (PVs) have been identified as common sources of ectopic foci leading to paroxysms of AF.^2,3^ However, in patients with (long-standing) persistent AF, arrhythmia-free survival after PVI is suboptimal due to a more extensive arrhythmogenic substrate.^1,4^ Therefore, ablation beyond the PVs, such as isolation of the left atrial (LA) posterior wall, the left atrial appendage (LAA), or the ligament of Marshall, can increase the success rate of these patients.^4^ But as with PVI, the success of these additional ablation strategies is dependent on achieving durable transmural lesions to ensure complete electrical isolation.^1^

Bipolar radiofrequency (RF)—referring to devices that apply energy on both sides of the tissue and thus differing from uniparietal bipolar and unipolar RF devices—are preferred given their enhanced capacity to create durable transmural lesions.^5,6^ The Cardioblate™ Gemini-S clamp (Medtronic, Galway, Ireland) uses pressurized saline-irrigated energy, allowing precise modulation of tissue heating and cooling by continuously adjusting its power output based on measured tissue impedance.^7^ However, it is currently unclear how to interpret the ablation curves when using this bipolar irrigated RF clamp during thoracoscopic AF ablation. As a result, the precise time point at which transmurality is achieved remains unknown, which increases the risk of underablation or overablation.

In this study, we aimed to analyze and describe the ablation curve dynamics observed during ablation procedures in pigs using the Cardioblate Gemini-S clamp. Based on these preliminary observations, we explored whether characteristic patterns in the ablation curves could be linked to lesion transmurality. Our intent is to contribute to a better understanding of curve interpretation, which may eventually support more accurate real-time assessment of lesion formation and reduce the risk of underablation or overablation. In addition, we retrospectively analyzed ablation curves in human patients who underwent hybrid thoracoscopic AF ablation using the same irrigated bipolar RF clamp, to explore whether similar or additional parameters could be identified that might be indicative of lesion transmurality.

## Methods

### Protocols

All animal care and procedures were carried out in accordance with the Danish Regional Animal Welfare Inspectorate (Miljø- og Fødevareministeriet, liscense #2018-15-0201-01608), animal welfare regulations, local regulations, and the recommendations of the Federation of European Laboratory Animal Science Associations (FELASA). All scientists and technicians involved have been accredited by the responsible ethics committee and corresponded to the ARRIVE guidelines in accordance with the directive guidelines 2010/63/EU of the European Parliament on the protection of laboratory animals.

### Animals and Preparation

In this study, 4 female healthy Landrace-Yorkshire-Duroc pigs, weighing ~50 kg and aged ~16 weeks old, were included. Animals were anaesthetized with propofol (15 µg/kg/h, Propolipid, Fresenius Kabi AB, Uppsala, Sweden) and fentanyl (5 µg/kg/h, Hameln Pharma GmbH, Hameln, Germany), intubated, and ventilated (tidal volume 10 mL/kg, frequency 14/min). Vascular access was established via the right jugular or femoral vein. An Arrow Seldinger 22-gauge arterial catheter (Teleflex, Wayne, PA, USA) was inserted into the femoral artery to monitor arterial blood pressure and periodically measure arterial blood gases (ABL90 FLEX, Radiometer, Copenhagen, Denmark). Pigs were placed in a supine position.

### Experimental Design

#### Ablation device

In this study, all ablations were performed using the Cardioblate Gemini-S clamp. This flexible irrigated RF clamp employs 2 rigid slim-line clamp jaws with an incorporated 6.3 cm active electrode on each jaw (Fig. 1a). During human isolated thoracoscopic and hybrid thoracoscopic AF ablation, the clamp is introduced via bilateral thoracoscopic access (Fig. 1b), and the jaws are positioned on either side of the tissue in the transverse and oblique sinuses to create a uniform current density to achieve sufficient heating across the clamped tissue in a continuous U-shaped lesion (Fig. 1c).

**Fig. 1.**
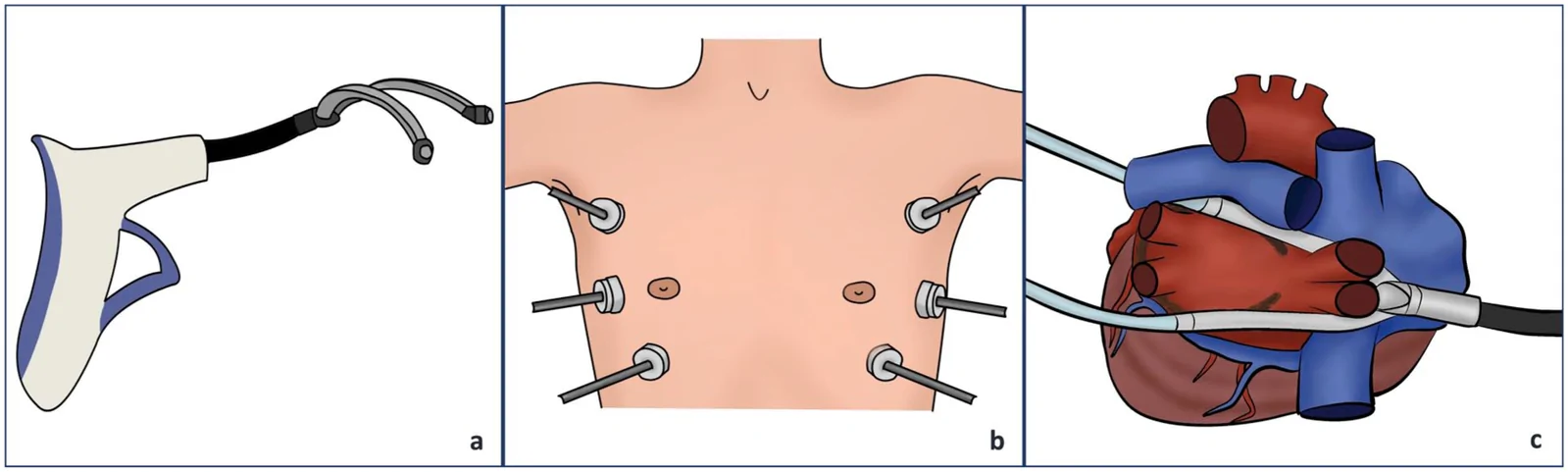
Thoracoscopic ablation with the bipolar pressurized saline-irrigated radiofrequency clamp. (a) The Cardioblate™ Gemini-S clamp (Medtronic, Galway, Ireland) with 2 rigid slim-line clamp jaws. (b) The clamp is introduced via bilateral thoracoscopic access, (c) with each jaw of the clamp being positioned on either side of the tissue in the transverse and the oblique sinus to create a uniform current density to achieve sufficient heating across the clamped tissue in a continuous U-shaped lesion.

The Cardioblate closed-loop dose-response algorithm measures tissue impedance (5 times per second), calculates impedance changes over time, and subsequently adjusts the power delivery level by automatically incrementing or decrementing the amount of energy delivered during an ablation cycle (power output operates between 15 and 55 W). Power delivery is increased in a stepwise fashion when impedance changes are still declining or reside in a flattening plateau, indicating tissue is still being ablated. Energy delivery is stepwise decreased as a response right after a certain rise (small, medium, large) in impedance is detected by the algorithm, to prevent overheating between the tissue and electrode interface, and to maintain sufficient energy delivery to the deeper tissue layers. This comprehensive approach not only ensures effective lesion formation but also minimizes the likelihood of thermal damage to the surrounding tissues and structures. Therefore, excessive rises in impedance need to be prevented to be able to create deep, consistent, and full-thickness transmural lesions.^8^

The Cardioblate automatic dose-response algorithm labels an ablation as “transmural” when certain criteria are met such as ablation duration, changes in energy output, and corresponding impedance drop during RF energy delivery. This is indicated by the generator as a vertical green line.

#### Ablation protocol in pigs

Sternotomy was performed, followed by opening of the pericardium. Small incisions were made at various locations on the atria and ventricles. A purse-string suture was placed around each incision to control bleeding during the procedure. One jaw of the Cardioblate Gemini-S clamp was positioned endocardially, and the other jaw was positioned epicardially, enabling real-time beating-heart endocardial-epicardial ablations (Fig. 2). Both atrial and ventricular tissue were selected for ablations to assess ablation behavior across different tissue types.

**Fig. 2.**
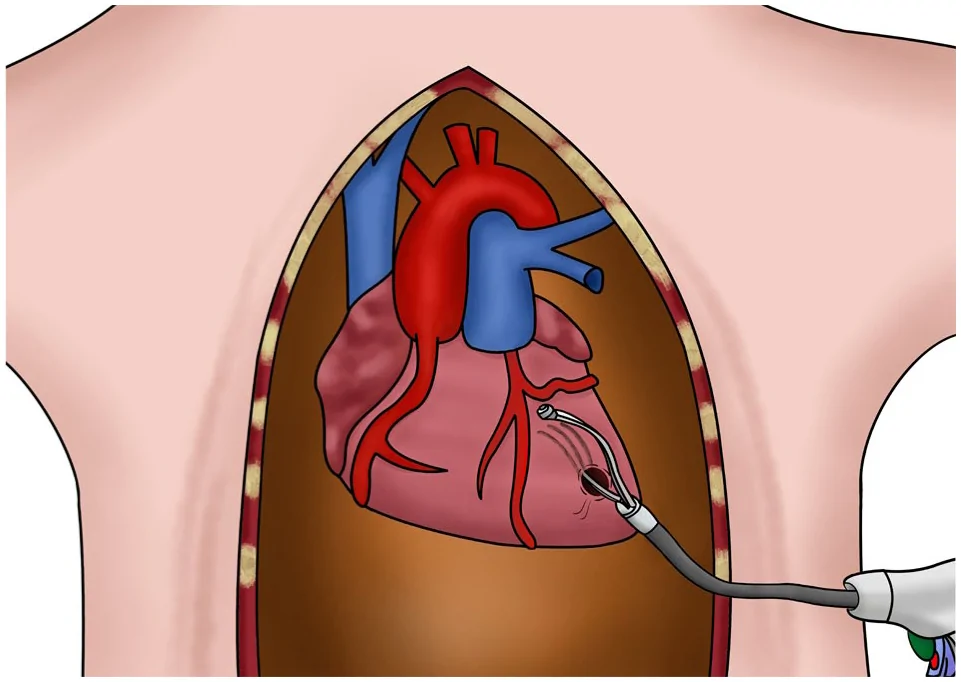
Ablation protocol in pigs. Frontal view of a pig’s heart, with an incision on the left ventricle, which is secured with a purse-string suture. The Cardioblate™ Gemini-S clamp (Medtronic, Galway, Ireland) is positioned with 1 jaw placed endocardially and the other jaw epicardially. Three ablation lines are visible, representing completed sites, while a fourth ablation is actively being performed.

The number of ablations per chamber (atrium or ventricle) ranged from 1 to 4, depending on the available tissue. At the first site of 1 chamber, a single ablation cycle was performed. If more tissue was available, a second site was used, where 2 ablation cycles were performed. If additional tissue allowed, a third site was incised, with 3 ablation cycles being performed. This process continued up to a maximum of 4 sites per chamber, with 4 ablation cycles at the fourth site (Fig. 2). For each new site, the clamp was repositioned, and the ablation procedure was repeated, with the clamp being opened and closed between each ablation cycle.

#### Identifying typical distinctive patterns of lesion transmurality in pigs

The first primary objective of this study was to identify typical distinctive patterns of lesion transmurality in ablation curves using the Cardioblate Gemini-S clamp. The ablation protocol was performed in the initial 3 pigs. Each ablation curve was photographed and stored for retrospective analysis.

#### Lesion assessment in pigs

After completing the ablation procedure, the pigs were sacrificed under full anesthesia, and the heart was excised directly after the last ablation cycle. Each chamber was carefully dissected, and a suture was placed on the right and cranial side of each chamber to ensure proper orientation for subsequent analysis (Fig. 3a). The chambers were rinsed with a sodium chloride solution to remove any thrombi remaining within the trabeculae. Subsequently, the chambers were immersed in 2,3,5-triphenyltetrazolium chloride (TTC) staining to macroscopically assess the tissue viability, facilitating the differentiation between metabolically active and inactive tissue; this redox indicator stains viable tissue red, whereas the nonviable tissue remains white. The samples were incubated in an oven at 36 °C for 90 min (Fig. 3b). Following staining, the chambers were longitudinally sectioned along the ablation lesion (Fig. 3c). By macroscopic evaluation, there was a differentiation of transmural versus nontransmural lesions. Macroscopic evaluation was performed to distinguish the transmural from the nontransmural lesions. Photographs were taken of the cross-sectioned ablated tissue to document the findings.

**Fig. 3.**
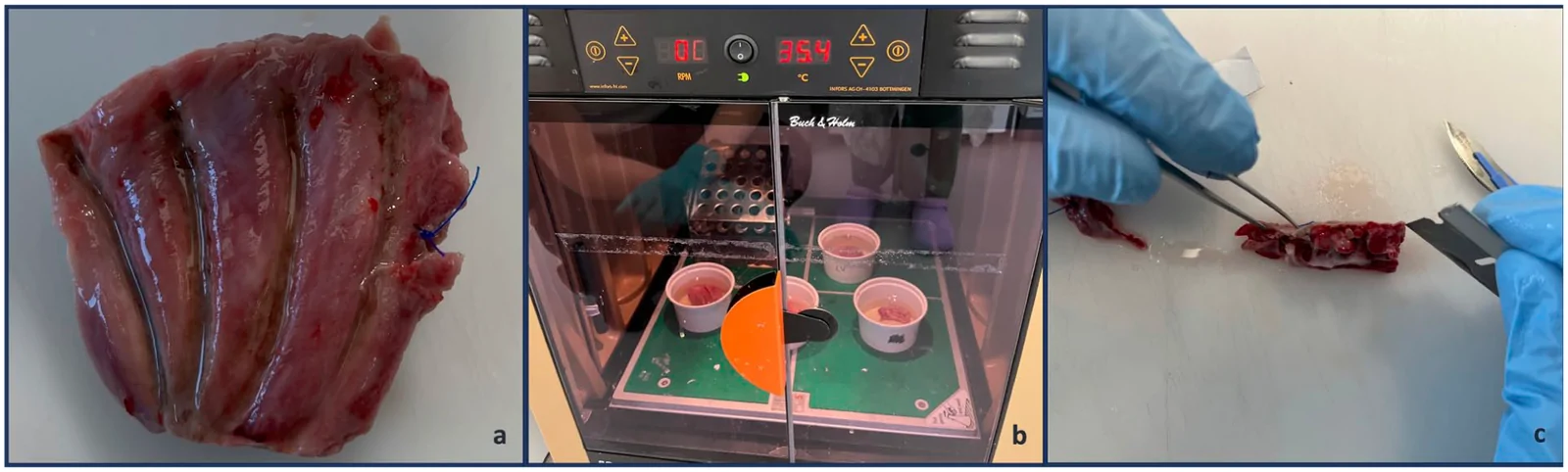
Macroscopic evaluation of ablated tissue in pigs. (a) Ventricular tissue with 4 ablation lines visible on the epicardial side, with the blue suture on the right side. (b) Incubator oven. (c) Longitudinally sectioned tissue of the left atrium. Red tissue in the middle indicates viable tissue; white-colored tissue at the most proximal part of the tissue indicates nonviable tissue.

The corresponding ablation curves were then retrospectively analyzed to identify typical distinctive patterns associated with lesion transmurality, which were confirmed with macroscopic evaluation.

#### Validation of typical distinctive patterns of lesion transmurality in pigs

In the fourth pig, ablations were performed on the 4 heart chambers at different sites and evaluated in real time. The goal was to perform multiple ablation cycles, until 1 of the identified typical distinctive transmurality patterns was observed in the ablation curves in real time. Once a typical pattern was identified in the ablation curve, the ablation at that site was stopped. The pig was then euthanized under full anesthesia, and the heart was excised for TTC staining and subsequent macroscopic analysis. This analysis was performed to validate whether the typical distinctive patterns in the ablation curves accurately predicted transmurality of the tissue, which was confirmed by macroscopic evaluation.

#### Transmurality indicators in human ablation curves

The second primary objective was to retrospectively analyze ablation curves from thoracoscopic hybrid AF ablation procedures, performed with the Cardioblate Gemini-S clamp, aiming to identify parameters indicative of lesion transmurality (Fig. 4, upper curve). The thoracoscopic hybrid AF procedure was performed in a single stage: epicardial ablation of the PVs and box lesion was carried out via thoracoscopy, followed within the same procedure by an endocardial electroanatomical voltage map via transvenous access. In addition to voltage mapping, the entrance and exit block were tested endocardially within all PVs and the box, with additional touch-up or additional endocardial ablation.

**Fig. 4.**
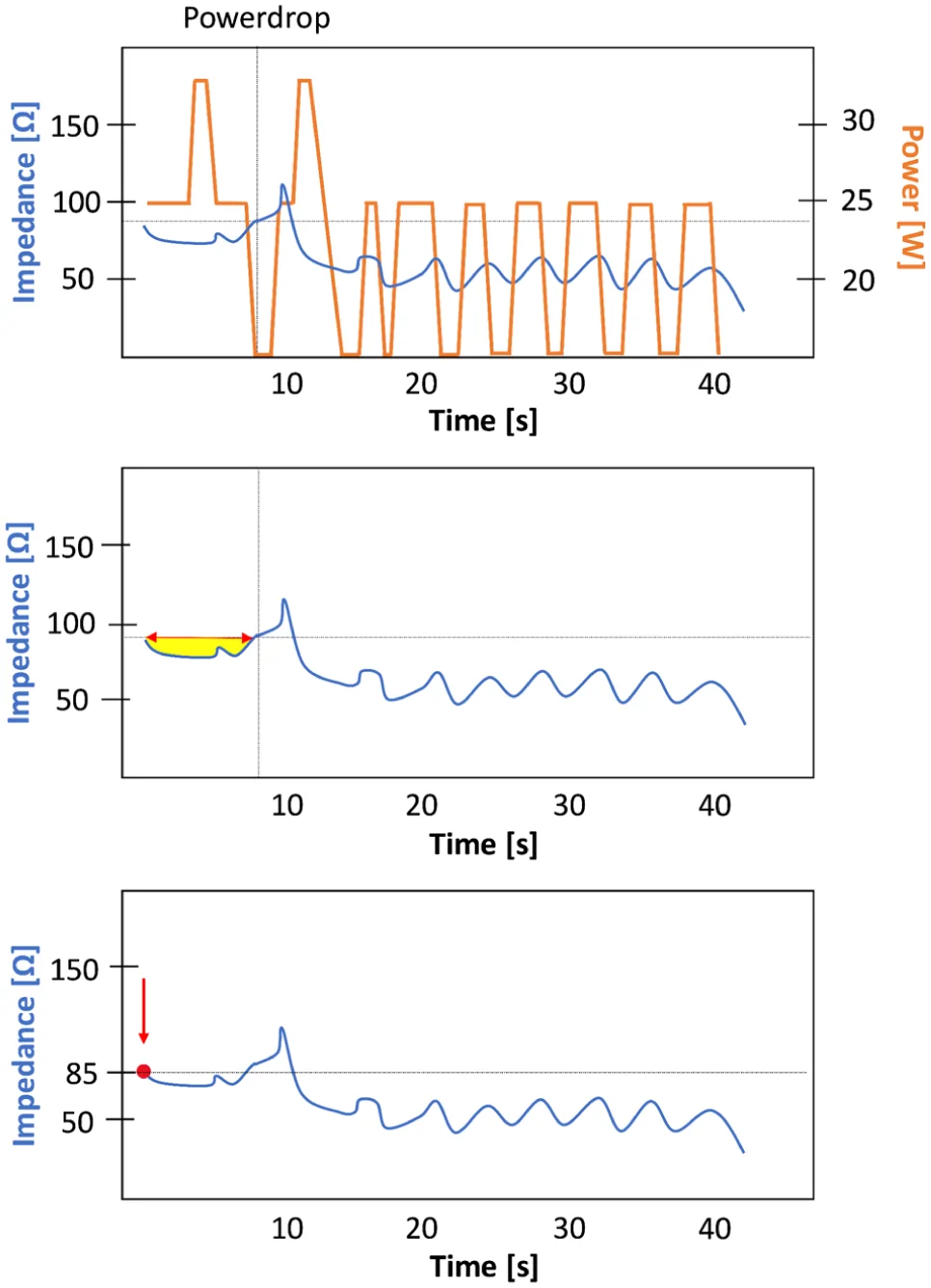
Ablation curve of 1 ablation cycle performed with the Cardioblate™ Gemini-S (Medtronic, Galway, Ireland) bipolar saline-pressurized irrigated radiofrequency clamp. The second curve shows the “bathtub” (yellow), indicating the amplitude of the “bathtub,” ranging from the onset impedance to the power drop (red arrow in the second graph). The third graph indicates the onset impedance (red arrow in the third graph).

The following retrospective analyses were conducted to evaluate transmurality in human ablation curves. These parameters were designed based on theoretical hypotheses regarding lesion formation and tissue response.

1. Bathtub analysis: the area under the curve (AUC) of the impedance curve, spanning from the onset impedance to the point of the initial substantial power drops, was quantified for each successive ablation in every patient (Fig. 4, middle curve). This method, termed the “bathtub” analysis, derives its name from the characteristic shape of the resultant curve, resembling a bathtub. The hypothesis suggests that with each successive ablation, the quantification of the AUC could unveil decreasing tissue resistance, thereby indicating an advancement toward achieving complete transmural lesions.
2. Onset impedance: for each consecutive ablation, precise measurements of the onset impedance were recorded and analyzed for every patient (Fig. 4, lower curve). The onset impedance could serve as a parameter in assessing the initiation of tissue heating and subsequent lesion formation during the ablation process. Associations between onset impedance values and transmurality outcomes were examined to elucidate potential predictive factors for transmurality.
3. Maximum impedance change analysis: the first derivative of impedance rise or fall for each ablation performed in every patient was calculated. The analysis enabled the characterization of dynamic impedance changes throughout the ablation, potentially offering insights in underlying mechanisms governing the transmural extent of ablation lesions by identifying patterns or correlations within the maximum impedance change data.

#### Retrospective analysis of typical distinctive patterns in human ablation curves

Finally, 3 typical distinctive patterns associated with lesion transmurality, as identified in the pig ablation experiments, were retrospectively evaluated in the human ablation curves. Ablation curves were independently reviewed by 2 investigators (B.M. and L.A.). For each patient, ablation curves for both left-sided and right-sided procedures were separately displayed and analyzed. This analysis aimed to determine whether the typical pattern indicators observed in the animal model could also be identified in the human data.

### Statistical Analysis

Study findings were analyzed and presented using descriptive statistics. Presented data for categorical variables included the number in each category; for continuous variables, the results included means and standard deviations and were compared using one-way analysis of variance and Tukey’s pairwise test, as appropriate. Data were analyzed using IBM SPSS Statistics, Version 26.0 (IBM Corp., Armonk, NY, USA).

## Results

### Typical Distinctive Patterns of Lesion Transmurality

Three typical ablation curve patterns, indicating transmurality, were described in the ablation curves of in the pigs, as seen in Figure 5:

- Type I: a sharp impedance rise (blue peak)
- Type II: 3 power drops with more than 1 energy level decline in 1 ablation curve (energy level decline: the Cardioblate Dose Response Algorithm automatically adjusts the energy output level [in Watts] based on the measured impedance change after the energy output adjustment. Impedance is measured 5 times per second. Based on the measured change in impedance [delta impedance over delta time] the generator adjusts the energy output by 1, 2, or 3 downward steps, until the lowest energy output level of 15 W is reached)
- Type III: a first power drop before 11 s with subsequent hovering of power levels between 15 and 25 W

**Fig. 5.**
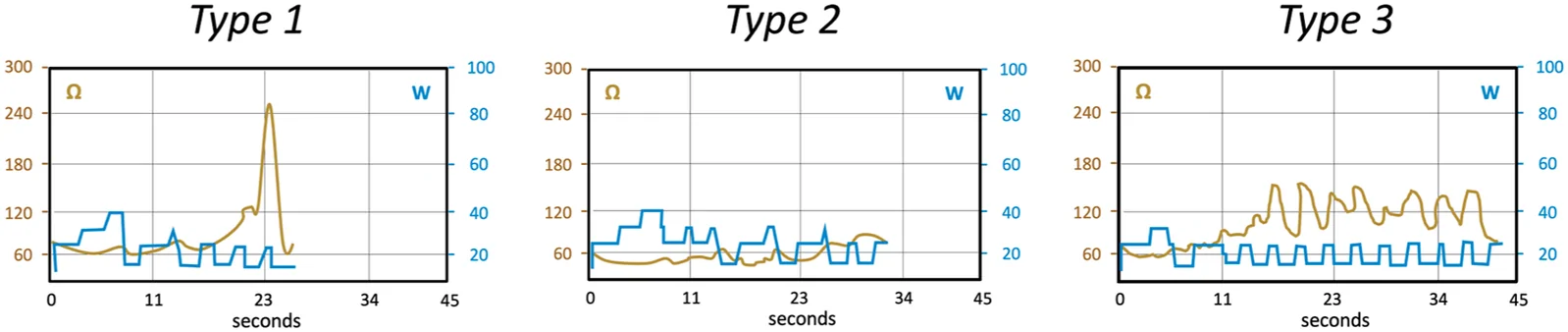
Typical distinctive patterns indicating transmurality. The blue curve represents the measured impedance (Ohms) of the tissue between the clamp jaws. The orange curve represents the amount of energy delivered to the tissue (Watts) in response to the measured impedance. Type 1: Sharp impedance rise (blue peak). Type 2: Three power drops with at least a 2-energy-level decline in 1 ablation curve. Type 3: Power drop of at least 2 energy levels and subsequent hovering of energy delivery between 15 and 25 W.

In the first 3 pigs, a total of 20 ablations were performed, with 50% (*n* = 10) considered transmural. In 80% of the transmural lesions, 1 of the 3 types of ablation curves was observed. Among the transmural lesions, 50%, 10%, and 20% corresponded to type I, II, and III indicators of transmurality, respectively, with 20% showing curve dynamics that did not exhibit typical ablation curve patterns.

In the lesions classified as not transmural (*n* = 10), no type I, II, or III indicators of transmurality were observed. In the fourth pig, all lesions (*n* = 7) were considered transmural based on macroscopic evaluation, comprising 86% type I and 14% type III indicators of transmurality (Fig. 6).

**Fig. 6.**
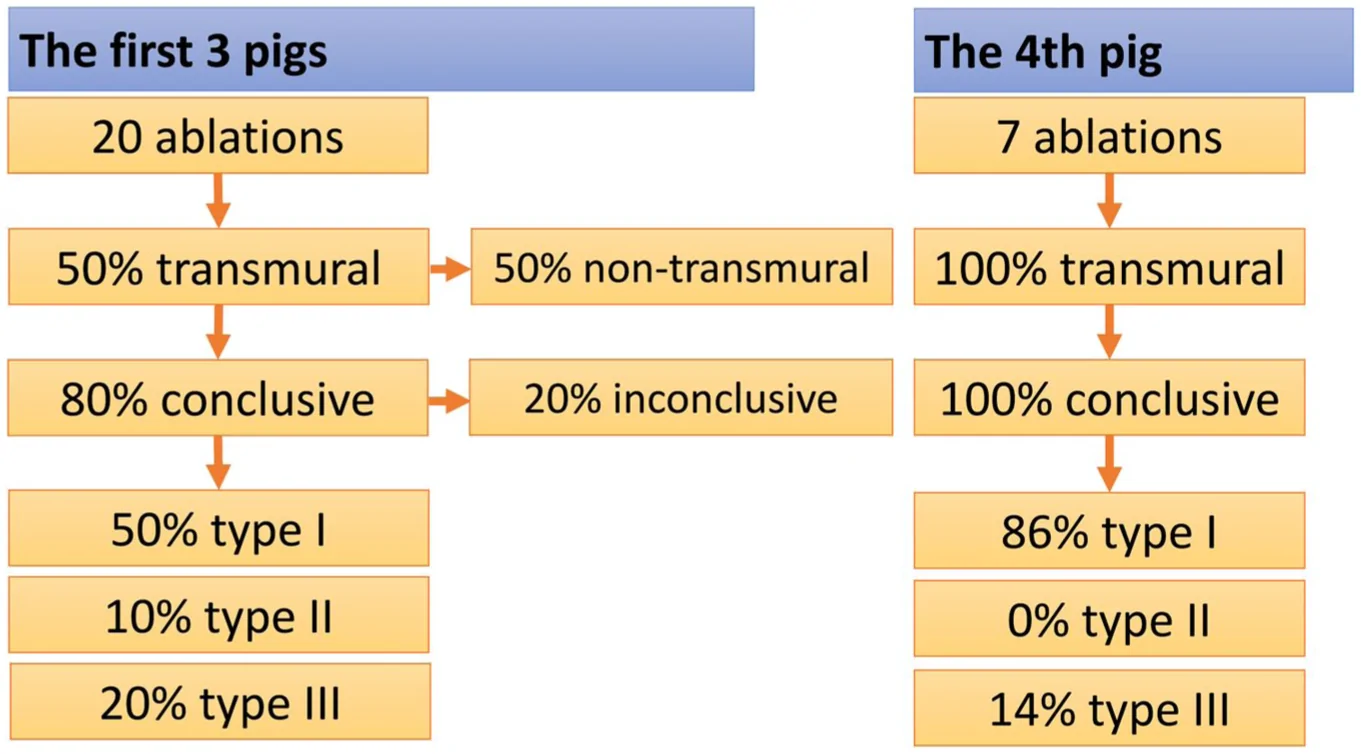
Flowchart of transmurality lesions in pigs. In the first 3 pigs, 20 ablations were performed, with 50% (*n* = 10) considered transmural. Among transmural lesions, 50%, 10%, and 20% were classified as type I, II, and III, respectively, with 20% inconclusive. Nontransmural lesions (50%) showed no indicators. In the fourth pig, all lesions (*n* = 7) were transmural, comprising 86% type I and 14% type III indicators.

### Transmurality Indicators in Human Ablation Curves

In the retrospective analysis of the human ablation curves, the point of transmurality based on the first occurrence of a typical pattern observed in the ablation curve was assessed. No discernible patterns were identified in terms of the “bathtub” for both left-sided and right-sided ablations (Fig. 7, Fig. 8).

**Fig. 7.**
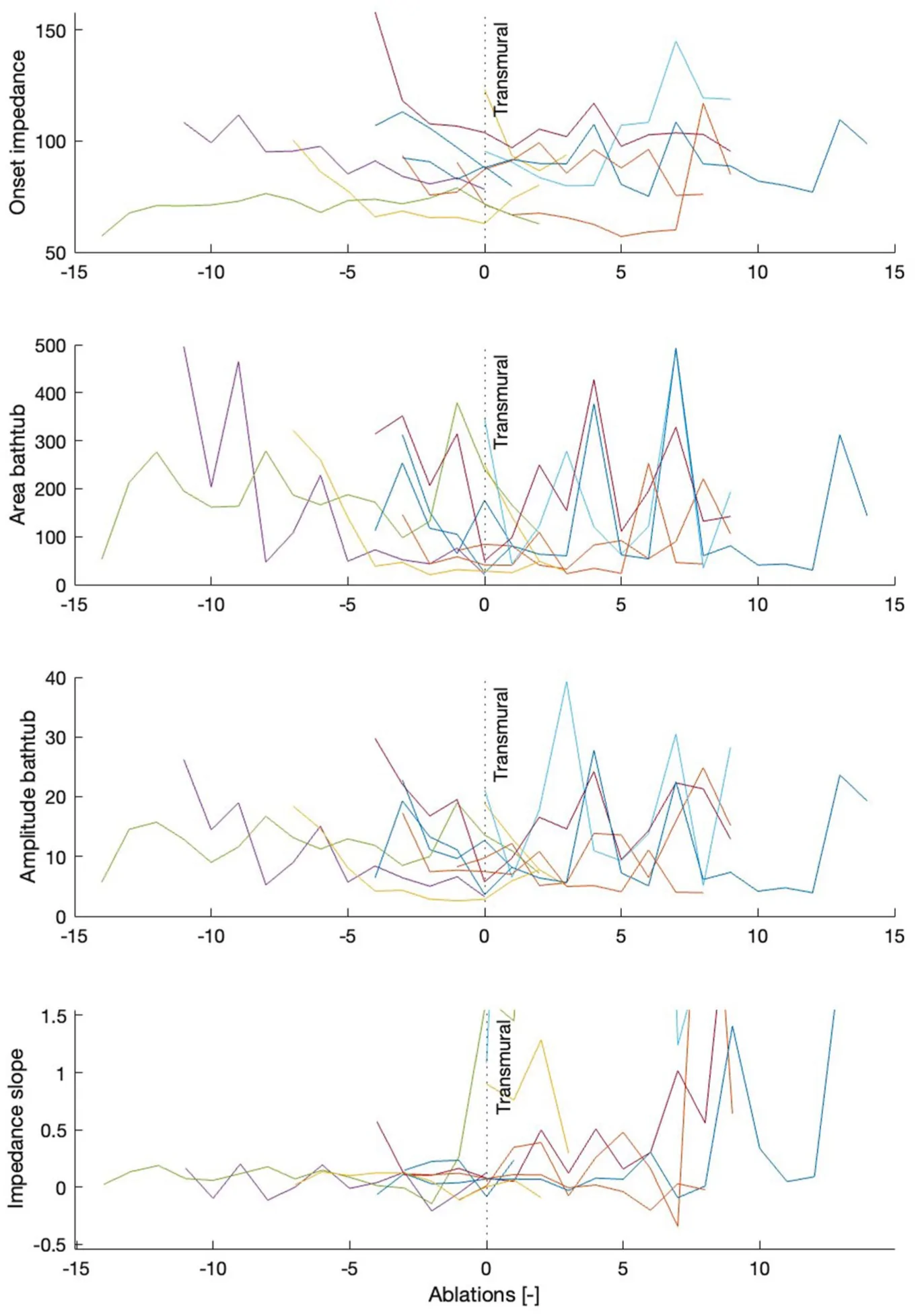
Left-sided ablations in humans. (a) Onset impedance. (b) Area under the bathtub curve. (c) Amplitude of the bathtub curve. (d) Impedance slope. The *x*-axis represents the number of ablations, with “0” indicating transmurality based on retrospective data analysis.

**Fig. 8.**
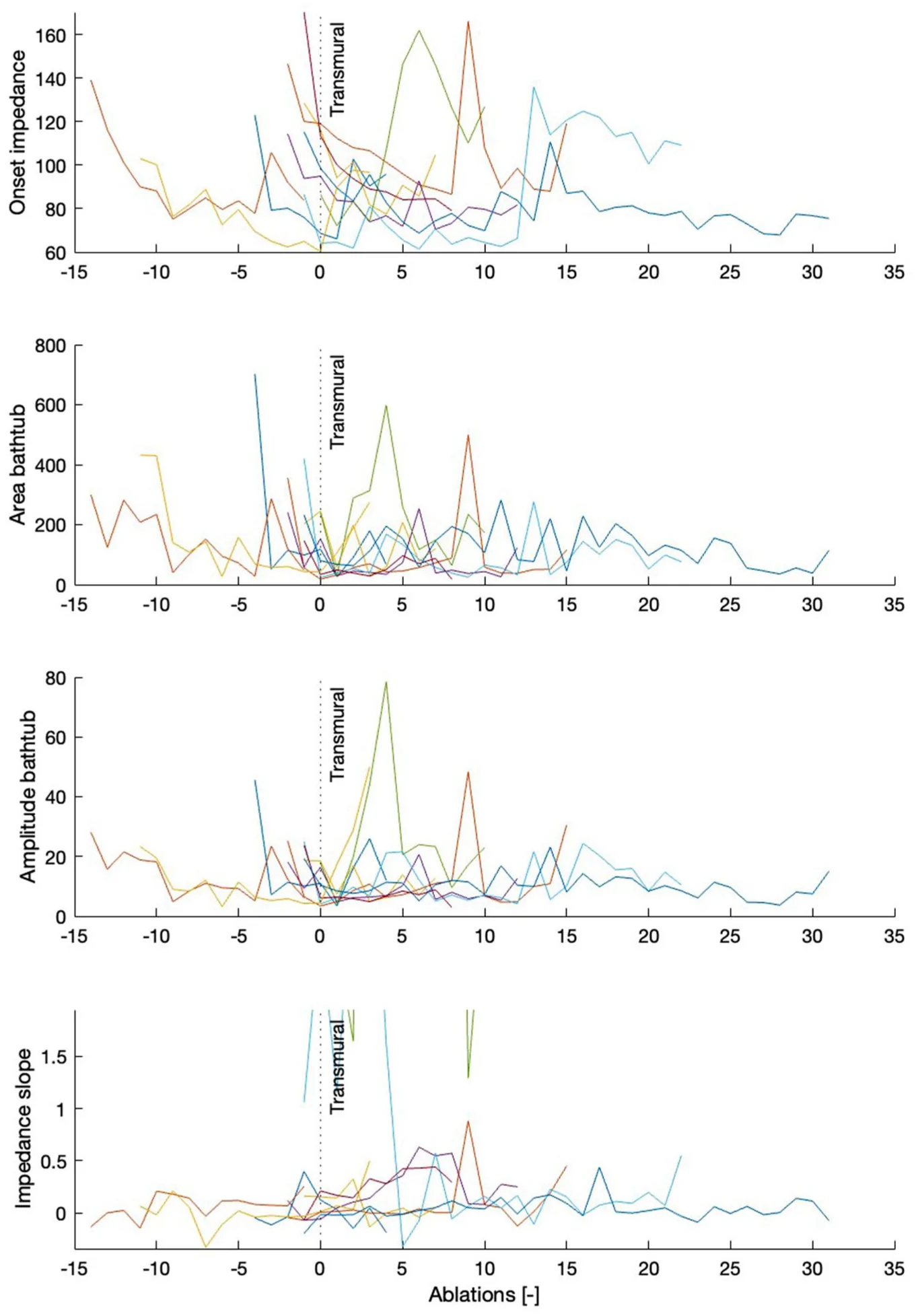
Right-sided ablations in humans. (a) Onset impedance. (b) Area under the bathtub curve. (c) Amplitude of the bathtub curve. (d) Impedance slope. The *x*-axis represents the number of ablations, with “0” indicating transmurality based on retrospective data analysis.

With each consecutive ablation, there was no consistent increase or decrease in the AUC, both in terms of surface area and amplitude of the respective impedance curve. Regarding the onset impedance, a visual trend was noted, as the onset impedance appeared to decrease progressively with each consecutive ablation until transmurality was achieved. This trend was observed in both left-sided and right-sided ablations. However, no association could be established between the absolute or relative decrease in onset impedance and the point at which transmurality was achieved. For the maximum impedance change analysis, there was no discernible pattern identified for both left-sided and right-sided ablations.

### Analysis of Typical Distinctive Patterns of Transmurality in Human Ablation Curves

In 11 patients, a bilateral thoracoscopic 1-stage hybrid AF ablation was performed at Medical University Center Maastricht. All patients received PVI with box lesion in 2 continuous U-shaped lesions with the Cardioblate Gemini-S clamp. Following the surgical epicardial ablation procedure, transvenous endocardial validation was conducted. All lesions demonstrated both endocardial exit and entrance block, confirming the transmurality of the lesions.

A total of 272 epicardial ablations were performed, with 41.9% (*n* = 114) ablated from the left side and with 58.1% (*n* = 158) via the right side. For the ablation cycles performed on the left side, 1 of the 3 typical distinctive patterns indicative of transmurality was retrospectively observed after a mean of 6 ± 4.46 ablation cycles. Following the identification of such an indicator, a mean of 5 ± 3.45 ablation cycles were performed. On the right side, 1 of the 3 distinctive indicators was observed after a mean of 3 ± 3.20 ablation cycles. After identifying the indicator, a mean of 12 ± 5.83 additional ablation cycles were performed. Of the 272 ablations, 46.3% (*n* = 126) were classified as transmural based on the 3 typical patterns of transmurality identified in the pig experiments. Among these transmural lesions, 30.2% corresponded to a type I pattern, 4.0% to a type II pattern, and 65.8% to a type III pattern.

## Discussion

This study offers preliminary insights into the use of the Cardioblate Gemini-S bipolar irrigated RF clamp by describing recurring ablation curve patterns. In an experimental porcine model, 3 typical patterns of transmurality were described in pigs: type I, sharp impedance rise (blue peak); type II, 3 power drops with at least a 2-energy-level decline in 1 ablation curve; and type III, power drop of at least 2 energy levels and subsequent hovering of energy delivery between 15 and 25 W. Second, in a retrospective analysis of human ablation curves during thoracoscopic hybrid AF ablation, the characteristic patterns were not only seen in the experimental model but were also consistently identified in human ablation curves, underlining their robustness as potential indicators of transmurality.

These findings should not be interpreted as a real-time simulation of clinical ablation but rather as a conceptual exploration of curve morphology under experimental conditions. The primary aim was not to validate a predictive technique but to identify recurring patterns that may reflect lesion development. Although these observations are preliminary and exploratory in nature, they suggest a potential relevance of these patterns for future clinical interpretation. However, further validation is required before any conclusions can be drawn regarding their predictive value in real-time clinical practice.

Transmural lesions of the PVs and the box lesion created during AF ablation are defined by the depth and completeness of the lesion, which are essential for achieving electrical isolation of the targeted tissue.^9^ Achieving transmurality of the lesions is crucial for optimal treatment outcomes, as it prevents arrhythmogenic triggers from exiting these areas and results in mass reduction.^10^ Tissue impedance plays a critical role in this process. During ablation, heating of the tissue causes a decrease in electrical impedance. This decline results from increased ion mobility and the denaturation of cell membrane proteins. Changes in cellular mechanisms responsible for maintaining osmotic gradients contribute to the progressive reduction in electrical impedance and therefore further facilitate lesion formation.^11,12^

During the ablation procedure, the user is able to see both energy output and impedance measurements in an overlapping graphical interface. Through this graphical display, the surgeon is able to recognize the typical ablation curve patterns identified in this study. These patterns allow the surgeon to predict when the ablation lesion is transmural and therefore when to stop ablating. In this way, unnecessary and limited or excessive applications can be avoided.

It is unclear how many applications are required to achieve transmurality of the ablated lesion using the bipolar irrigated RF clamp. Although experimental models have shown that a single ablation using a bipolar clamping device can create transmural lesions,^4^ our clinical experience suggests that multiple applications are often necessary to achieve exit and entrance block endocardially during a thoracoscopic hybrid AF ablation.

The graphical interface of the Cardioblate Gemini-S clamp generator displays a vertical green “transmurality” line after each ablation when its automatic dose-response algorithm classifies the lesion as transmural. This classification is based on specific technical criteria such as ablation duration, changes in energy output, and corresponding impedance drop during RF energy delivery. However, our findings in the porcine model demonstrate that, despite the device consistently indicating transmurality according to these parameters, histological analysis does not always confirm true transmural lesions. In line with this, the apparent mismatch between predicted transmurality and acute endocardial block likely reflects the distinction between acute lesion formation and durable conduction block, as late reconnection is well-described once edema and acute thermal injury subside.

This discrepancy may be due to several factors, such as epicardial fat, fibrosis, and increased atrial wall thickness.^10^ Epicardial fat is characterized by a high electrical resistance and can therefore limit RF current penetration, which reduces the effectiveness of ablation.^13^ Similarly, thicker atrial walls present additional challenges to achieving full lesion depth. To address this, the experimental ablation protocol included both atrial and ventricular tissue in pigs. Atrial tissue in pigs is notably thin compared with human atrial tissue, making it a challenging but useful model for testing. Ventricular tissue, with a thicker structure, provided additional data to analyze ablation across different tissue thicknesses.

Another recent study by Yates et al. concluded that a single ablation on human atrial tissue with an irrigated bipolar RF clamp was insufficient to create transmural lesions. This study was conducted in an ex vivo human heart model, and a Cox maze IV procedure was performed using the Cardioblate device.^13^ The study findings demonstrated a significant improvement in lesion transmurality when performing 2 successive ablations compared with a single ablation (74% vs 92%, *P* = 0.017), particularly in regions characterized by higher adiposity, such as the PVs. The lesion width was significantly less (1.51 vs 1.85 mm, *P* < 0.001) and the ablation depth was significantly deeper (1.27 vs 0.78 mm, *P* < 0.001) in the transmural lesions compared with nontransmural lesions, underscoring the importance of tissue characteristics in determining ablation efficacy. It is important to note that irrigated RF differs fundamentally from dry RF; whereas in dry RF continuous clamping without reopening is preferred to maintain consistent energy delivery, in irrigated RF it is beneficial to briefly open the clamp between consecutive applications to promote membrane irrigation and prevent overheating.

In contrast to clinical practice, in which epicardial–epicardial ablation is often feasible, epicardial–endocardial ablation was performed in the experimental part of the study. This approach was chosen for 2 reasons. First, anatomical constraints played a role; the Cardioblate Gemini-S clamp is relatively long compared with the small porcine atria, making epicardial–epicardial positioning technically unfeasible. Second, the atrial wall in pigs is very thin, which makes transmural ablation achievable with an epicardial–endocardial or endocardial–epicardial approach. This also aligns with the human clinical setting, in which atrial ablation is typically performed using similar strategies. In contrast, for the ventricles—in which the myocardial wall is significantly thicker—an endocardial-to-epicardial approach is more appropriate and effective. It is important to emphasize that the primary objective of this study was not to evaluate the clinical applicability of this clamp in a porcine model but rather to explore and characterize ablation curve dynamics in different tissue contexts. Therefore, the experimental design was tailored to facilitate the analysis of curve morphology, rather than to replicate clinical ablation protocols.

In 20% of the transmural curves, no typical ablation curve pattern was observed. These 20% of curves reflect the absence of typical ablation curve patterns in 2 ablation applications, where transmurality was present on macroscopic evaluation. As such, it might be that lesions are already transmural before the typical ablation curve pattern is observed. But much more importantly, every time a defined typical ablation curve pattern is present, transmurality can be assumed with certainty. This interpretation is further supported by the second experimental protocol (the fourth pig), in which ablations were continued until a typical ablation curve pattern was seen. In this setting, the presence of such a typical ablation curve (e.g., type I, type II, or type III) was associated with transmurality in 100% of cases.

In addition, the bathtub analysis revealed no clear patterns, underscoring the complexity of the tissue response during ablation and the multifactorial nature of transmurality. Contrary to the initial hypothesis that the AUC would decrease with each ablation, this was not observed, indicating a more complex interplay of factors. Also, although visual inspection suggested a decrease in onset impedance as transmurality was achieved, further quantitative analyses are needed to confirm this observation and investigate the underlying mechanisms in larger patient populations.

In the 11 patients investigated in this study, the 3 types of patterns identified in the experimental setting were also observed in clinical ablations. Retrospective analysis based on these 3 indicators showed that, on average, transmurality was achieved after 6 applications on the left side and 3 applications on the right side. This suggests that, after achieving transmurality, an average of 5 unnecessary applications are performed on the left and 12 on the right side. These findings highlight the potential to optimize ablation protocols and reduce unnecessary energy delivery. This optimization can lead to shorter procedure times, lower the risk of damage to adjacent structures, and decrease the occurrence of microemboli. Furthermore, the distribution of transmural lesions among different indicator types highlighted the heterogenicity of lesion characteristics. A limitation of this study is the small sample size and the lack of data on epicardial fat or atrial wall thickness, which may influence the number of ablations needed for transmurality. This highlights the need for further research with a larger patient group.

The findings from this experimental and translational study provide preliminary insights that may support the interpretation of human ablation curves with regard to lesion transmurality. Although this study was not designed to establish clinical applicability, the identification of recurring curve patterns offers a potential starting point for recognizing objective indicators that could assist in intraoperative assessment. If consistently observed in broader patient populations, these patterns may eventually contribute to more standardized approaches to evaluating lesion quality during thoracoscopic AF ablation using the Cardioblate clamp. However, further translational research is required to validate these preliminary indicators in clinical settings and to determine their actual utility in guiding ablation strategies and improving procedural outcomes.

### Limitations

This study has several limitations that should be considered when interpreting the results. First, the experimental component was conducted in a porcine model, which differs from the human atrial substrate in terms of tissue thickness, anatomical proportions, and presence of epicardial fat. These anatomical differences limited the feasibility of epicardial–epicardial clamping, leading to the use of an epicardial–endocardial ablation approach, which may affect lesion characteristics and energy delivery dynamics. However, the study was not designed to test or predict transmurality itself in the porcine model. Rather, the aim was to identify typical ablation curve patterns that occur when transmurality is achieved. Second, the number of both the pigs (*n* = 4) and human cases (*n* = 11) included in the retrospective analysis was limited, and we acknowledge that macroscopic assessment may overestimate lesion transmurality. Therefore, histological confirmation should be considered the gold standard. As such, the identification of curve patterns remains observational and exploratory in nature. No histological validation could be performed in this study, which represents a major limitation. Validation based on a single animal (the fourth pig) is insufficient; however, given that the irrigated RF clamp is already routinely used in clinical practice, clinical validation during thoracoscopic single-staged hybrid AF procedures is considered more appropriate and ethical than further animal experiments. Currently, a validation study in humans is being conducted to confirm the presence and diagnostic value of the identified typical ablation patterns. In these procedures, epicardial ablation is performed until typical ablation curve patters are observed, followed by immediate endocardial assessment of transmurality, enabling systematic validation of the typical ablation curve patterns in human subjects. This will include a larger patient population. Of note, same-day endocardial block after prior epicardial ablation is evidence of acute block only. A staged approach with a delayed endocardial map may provide a more accurate representation of durable block. Third, no direct clinical outcomes—such as long-term rhythm success or conduction block epicardial or endocardial—were correlated with the identified curve characteristics. Therefore, the potential clinical impact of these findings remains hypothetical at this stage. Finally, the use of an irrigated RF clamp with automated impedance-based energy modulation introduces variability that may differ from other devices, limiting generalizability.

## Conclusions

This study presents a conceptual framework for identifying objective indicators of lesion transmurality based on ablation curve dynamics, derived from experimental observations. These preliminary patterns were also observed in a small cohort of human ablation curves, suggesting their potential relevance for future clinical interpretation. Although the findings may point toward opportunities to optimize ablation protocols and reduce unnecessary energy delivery, no definitive conclusions can be drawn at this stage. Further research in larger and more diverse patient populations is needed to explore the consistency, reliability, and clinical utility of these curve-based indicators within surgical AF ablation therapy. Currently, a validation study in humans is being conducted to confirm the presence and diagnostic value of the identified ablation patterns.

## References

[1] Van Gelder IC Rienstra M Bunting KV , et al. 2024 ESC guidelines for the management of atrial fibrillation developed in collaboration with the European Association for Cardio-Thoracic Surgery (EACTS). Eur Heart J 2024; 45: 3314–3414. 10.1093/eurheartj/ehae176 39210723

[2] Haissaguerre M Jaïs P Shah DC , et al. Spontaneous initiation of atrial fibrillation by ectopic beats originating in the pulmonary veins. N Engl J Med 1998; 339: 659–666. 10.1056/NEJM199809033391003 9725923

[3] Schotten U Verheule S Kirchhof P , et al. Pathophysiological mechanisms of atrial fibrillation: a translational appraisal. Physiol Rev 2011; 91: 265–325. 10.1152/physrev.00031.2009 21248168

[4] Maesen B Van-Loo I Pison L , et al. Surgical ablation of atrial fibrillation: is electrical isolation of the pulmonary veins a must? J Atr Fibrillation 2016; 9: 1426. 10.4022/jafib.1426 PMC5089514 27909520

[5] Matteucci F Maesen B De Asmundis C , et al. Comparison between biparietal bipolar and uniparietal bipolar radio frequency ablation techniques in a simultaneous procedural setting. J Interv Card Electrophysiol 2021; 61: 567–575. 10.1007/s10840-020-00852-5 PMC8376704 32833110

[6] Bugge E Nicholson IA and Thomas SP. Comparison of bipolar and unipolar radiofrequency ablation in an in vivo experimental model. Eur J Cardiothorac Surg 2005; 28: 76–80. 10.1016/j.ejcts.2005.02.028 15982589

[7] Nakagawa H Yamanashi WS Pitha JV , et al. Comparison of in vivo tissue temperature profile and lesion geometry for radiofrequency ablation with a saline-irrigated electrode versus temperature control in a canine thigh muscle preparation. Circulation 1995; 91: 2264–2273. 10.1161/01.cir.91.8.2264 7697856

[8] Melby SJ Gaynor SL Lubahn JG , et al. Efficacy and safety of right and left atrial ablations on the beating heart with irrigated bipolar radiofrequency energy: a long-term animal study. J Thorac Cardiovasc Surg 2006; 132: 853–860. 10.1016/j.jtcvs.2006.05.048 17000297

[9] Verheule S Eckstein J Linz D , et al. Role of endo-epicardial dissociation of electrical activity and transmural conduction in the development of persistent atrial fibrillation. Prog Biophys Mol Biol 2014; 115: 173–185. 10.1016/j.pbiomolbio.2014.07.007 25086270

[10] Hindricks G Potpara T Dagres N , et al. 2020 ESC guidelines for the diagnosis and management of atrial fibrillation developed in collaboration with the European Association for Cardio-Thoracic Surgery (EACTS): the Task Force for the diagnosis and management of atrial fibrillation of the European Society of Cardiology (ESC) developed with the special contribution of the European Heart Rhythm Association (EHRA) of the ESC. Eur Heart J 2021; 42: 373–498. 10.1093/eurheartj/ehaa612 32860505

[11] Simmers TA De Bakker JM Wittkampf FH , et al. Effects of heating on impulse propagation in superfused canine myocardium. J Am Coll Cardiol 1995; 25: 1457–1464. 10.1016/0735-1097(94)00559-9 7722149

[12] Haines DE and Watson DD. Tissue heating during radiofrequency catheter ablation: a thermodynamic model and observations in isolated perfused and superfused canine right ventricular free wall. Pacing Clin Electrophysiol 1989; 12: 962–976. 10.1111/j.1540-8159.1989.tb05034.x 2472624

[13] Yates TA McGilvray M Schill MR , et al. Performance of an irrigated bipolar radiofrequency ablation clamp on explanted human hearts. Ann Thorac Surg 2023; 116: 307–313. 10.1016/j.athoracsur.2023.02.055 36935027

